# Productivity Measurement through IMU-Based Detailed Activity Recognition Using Machine Learning: A Case Study of Masonry Work

**DOI:** 10.3390/s23177635

**Published:** 2023-09-03

**Authors:** Sungkook Hong, Youngjib Ham, Jaeyoul Chun, Hyunsoo Kim

**Affiliations:** 1 Department of Architectural Engineering, Dankook University, 152 Jukjeon-ro, Suji-gu, Yongin-si 16890, Gyeonggi-do, Republic of Korea; dk_hsk22@dankook.ac.kr (S.H.); jaeyoul@dankook.ac.kr (J.C.); 2 Department of Construction Science, Texas A&M University, 3137 TAMU, College Station, TX 7784, USA; yham@tamu.edu

**Keywords:** detailed activity classification, schedule management, inertial measurement unit (IMU), convolutional neural network (CNN), long short-term memory (LSTM), masonry

## Abstract

Although measuring worker productivity is crucial, the measurement of the productivity of each worker is challenging due to their dispersion across various construction jobsites. This paper presents a framework for measuring productivity based on an inertial measurement unit (IMU) and activity classification. Two deep learning algorithms and three sensor combinations were utilized to identify and analyze the feasibility of the framework in masonry work. Using the proposed method, worker activity classification could be performed with a maximum accuracy of 96.70% using the convolutional neural network model with multiple sensors, and a minimum accuracy of 72.11% using the long short-term memory (LSTM) model with a single sensor. Productivity could be measured with an accuracy of up to 96.47%. The main contributions of this study are the proposal of a method for classifying detailed activities and an exploration of the effect of the number of IMU sensors used in measuring worker productivity.

## 1. Introduction

### 1.1. Motivation of the Study

Construction jobsites are complex environments since multiple units comprising different workers and construction equipment operate simultaneously [1,2,3,4]. Numerous construction activities are mainly conducted by workers, which means that the labor productivity may be the key information for managing and scheduling the construction activities [5]. In the aspects of productivity that focus on worker’s activities, efforts have been made toward productivity monitoring and measurement (e.g., work sampling, direct observation, surveys/interviews). However, they are discontinuous, time-consuming, and labor-intensive [6,7,8]. Hence, the requirement for an automated method to measure productivity has increased.

Recent advancements in algorithms (e.g., machine vision) and hardware (e.g., visual sensor, GPU, etc.) have led to a high availability of vision-based approaches for activity classification [9]. Prior studies have demonstrated that vision-based methods can robustly detect worker activity in jobsites. Despite the benefits of these approaches, there is an underlying challenge associated with the use of fixed cameras for monitoring workers continuously when they are out of frame. Given the limited number of visual sensors in a jobsite, installing cameras in order that there are no blind spots, to monitor every activity of every worker, may not be feasible.

Research has also been conducted to predict workers’ status using other wearable sensors (e.g., photoplethysmography (PPG), electroencephalogram (EEG), electrocardiogram (ECG), and insole pressure sensor, etc.). ECG is highly advantageous as it can quickly determine the occurrence of cardiovascular diseases by measuring the electrical signals of the heart [10]. Prior studies have attempted to facilitate the determination of the workload of workers through their heart rate variability using PPG [11,12,13]. Additionally, EEG using brain waves has the advantage of being able to judge the various physiological states of workers [14,15]. In the case of insole sensors, studies have attempted to predict various safety accidents such as balance collapse through the distribution of loads applied to workers [16]. Although the above-mentioned wearable sensors have been applied for the improvement of safety in the construction industry, it is challenging to measure the workers’ activity at a detailed level using the above sensors.

### 1.2. Key Research Gaps and Hypothesis

Motion sensors such as IMUs have been used at construction jobsites due to their small size, low battery power consumption, and availability to function under low-light conditions [17,18]. These sensors are also highly durable in construction jobsites, where workers are frequently in contact with rigid structures [19]. Previous studies have been conducted on the classification of human motions using IMU sensor data [18,19,20,21]. Additionally, from a practical perspective (e.g., budget, computational load), it is important to reduce the number of attached sensors while maintaining analysis accuracy in field applications [22]. Generally, it is regarded that if more IMU sensors can be attached to a worker’s body, it is more likely to lead to a higher accuracy of activity classification [23]. However, investigating the impact of a high number of IMU sensors on the accuracy of activity classification is critical for field application. If an acceptable level of accuracy for measuring labor productivity can be achieved, a small number of IMU sensors can be used in field application. Notably, using fewer IMU sensors has the advantages of requiring a lower budget and alleviating discomfort caused by sensor attachment. 

In this regard, the basic hypothesis of this study is as follows: “Activity classification accuracy may vary based on the number of sensors and types of algorithms used. Furthermore, productivity measurement must be performed with desirable classification accuracy”. This study aims to investigate the feasibility of the framework that can automatically measure productivity based on activity classification. To achieve this, the authors collected accelerometer and gyroscope data from 30 masons during masonry work. 

### 1.3. Content Organization

The subsequent section outlines the specifics of the proposed method, the experimental designs, and the analysis of the results aimed at calculating the productivity and actual working time of each worker. This paper concludes by discussing the results and delving into the further contributions and implications of the findings.

### 1.4. Related Works and Research Background

Labor productivity is directly related to the profitability of construction projects owing to the labor-intensiveness of the construction industry [24]. Labor productivity does not consider the individual ability of the workers since the existing productivity measurement methods are based on the input production volume and the output volume [25]. In addition, in the construction industry, measuring the abilities of individual workers is challenging due to the extensive sites and the substantial manpower required [26]. Therefore, measuring individual activity is essential for efficient human resource management. 

In the aspects of activity measurement, several studies have employed various approaches to recognize activity (e.g., computer vision and wearable sensor-based technology). For example, Peddi et al. [27] proposed an algorithm that analyzed human pose in real-time using computer vision and machine learning for construction productivity. Another approach that analyzes construction activity by crowdsourcing has been used to recognize the activities of various workers [28]. Gong et al. [29] proposed a video interpretation method for productivity analyses, and Luo et al. [30] proposed a convolution neural network (CNN)-based framework that integrates red, green and blue (RGB), optical flow, and gray stream CNNs to accurately monitor worker activity. With the advancement of deep learning algorithms such as the CNN and wide-spread camera devices such as the closed-circuit television (CCTV), vision-based activity recognition approaches have been applied widely in various fields [1,31,32]. However, activity recognition can be carried out only when the worker is within the camera’s line of sight. It is challenging to detect the activity of the workers when they have their backs to the cameras. For example, masonry workers usually stack bricks from one wall to another wall or from one column to another column with their backs to the cameras. Even if the cameras are installed in front of them, the workers may be out of the camera’s line of sight as the bricks pile up. In this case, activity recognition using vision-based approaches is ineffective. 

However, wearable sensors such as IMU, EEG, PPG, ECG, and insole pressure sensors have been widely applied for the safety and health management of workers. Small EEG, PPG, and ECG devices can be employed and used to gather physiological data (such as the heart rate, heart rate variability, and brain wave) of workers without hindering their work [33,34,35]. Prior studies using insole pressure sensors have attempted to perform step counting, posture estimation, and activity recognition [36]. However, the current study aims to classify the detailed activities of workers for measuring individual labor productivity. Although sensor data from EEG, PPG, and ECG for obtaining the physiological status of the workers can represent their workloads and stress levels, these sensors cannot reflect their detailed body movements. Previous studies have shown that insole pressure sensors can only classify walking steps or simple postures such as standing or sitting [37]. 

IMU sensors attached to different body parts (such as both wrists, both ankles, and the head) can be used to reflect detailed body movements of workers. For example, Kim et al. [38] validated the technical and practical feasibility of using IMU sensors for motion detection by classifying six motions of workers (e.g., standing, bending-up, bending, bending-down, walking, and twisting) using the long short-term memory algorithm (LSTM). Joshua et al. [39] showed the potential for the automation of activity recognition in construction sites by comparing classifiers (Naïve Bayes (NB), decision trees (DT), and multilayer perceptron (MP)) and obtained approximately around 80% accuracy using IMU sensors attached to the waist. Moreover, prior studies have proposed methods to improve activity recognition by analyzing the movement of workers or construction equipment [7,40,41,42].

Since, at construction sites, the entire body of worker moves when they are at work, this study uses IMU sensors to recognize activities. Notably, the robustness and resilience of IMU sensors are appropriate for use in construction sites as compared to other sensors such as those based on vision [19]. Furthermore, motion sensors such as IMUs are not affected by low illumination conditions that are common in construction jobsites such as underground construction or night-time work [17]. Due to the above-mentioned advantages, IMU-based approaches have also been adopted in construction research including human activity recognition [43,44], equipment recognition [40,45,46], and safety risk detection [47,48]. Therefore, the IMU-based activity recognition was selected as the objective for this study. 

Previous studies have analyzed the activity of workers by extracting their movements using IMU sensors. Specifically, IMU-based sensing approaches were used for recognizing and classifying the activities of the workers. However, a gap remained to be addressed as to whether the activity classification accuracy was in agreement with the productivity accuracy. Therefore, in this study, we aim to (1) extract worker activities and productivities by analyzing the sensor data, and (2) compare these data with the actual productivity results (recorded by site managers) for validating the effectiveness of the proposed method. The following section presents the experiment design, and the algorithms are used in this study for analyzing worker movement using IMUs. 

## 2. Methodology

**Figure 1:**
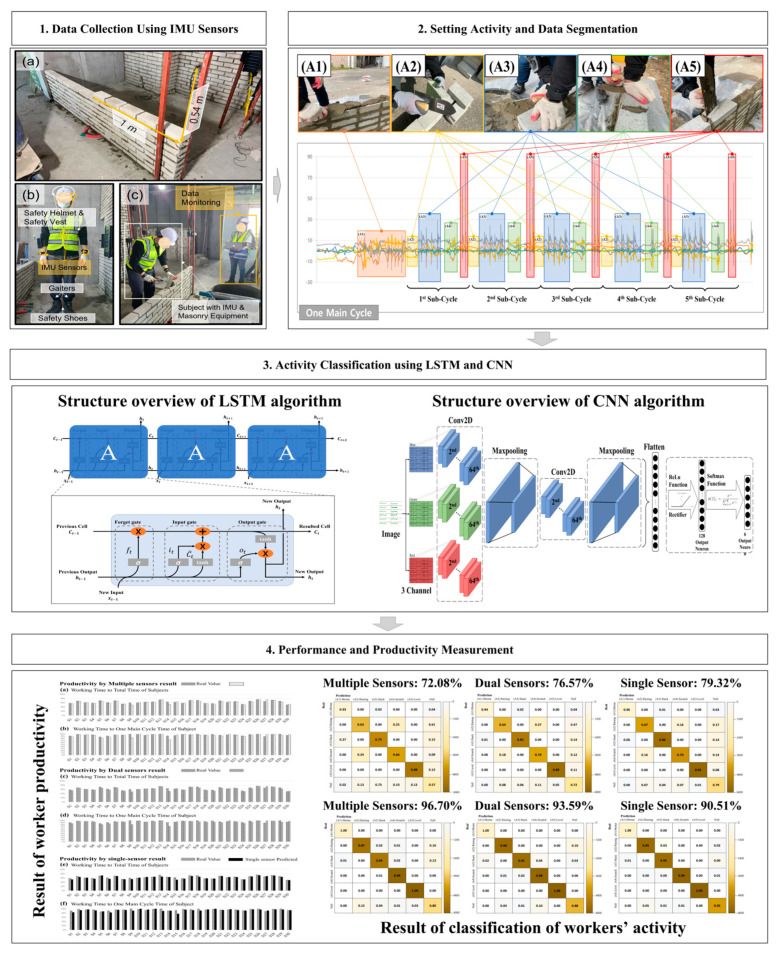
Research framework.

This study aimed to perform accurate activity classification using IMU sensor data and accurate productivity measurement based on the results of activity classification. In particular, the masonry process was selected for activity classification. As shown in Figure 1, workers were assigned to stack brick walls for the experimental period. Worker movement data were collected using IMU sensors (APDM Opal Movement Monitor) attached to both wrists, the back of the sternum, and the right ankle of each worker. Since the task of masonry involves the movement of the entire body, the sensors were attached all over each worker’s body. The sampling frequency of all the sensors was 128 Hz. Both sensors (accelerometer and gyroscope) had a bandwidth of 50 Hz. The collected data were transferred from the sensors to the main storage via Bluetooth low energy (BLE). To test the feasibility of activity classification using a reduced number of sensors, we analyzed the accuracy by decreasing the number of sensors (e.g., four sensors placed throughout the body, two sensors on both wrists, and one sensor on the dominant hand’s wrist). In addition, to minimize the influence of factors such as shaking (resulting from improper fixation) when capturing body movements, the sensors on the wrists were directly attached to the workers’ skin, and the one on the ankle was installed on the gaiters. The sensor on the back of the sternum was tightly attached to accurately reflect the movement of the upper body without distortion using a fixing belt. For data analysis, we employed (1) an LSTM, which utilizes the temporal sequence’s shape as a feature, and (2) a CNN, which interprets the data as an image by converting the time series data into image data. Hence, we derived the classification accuracy while using varying numbers of sensors during the training of the LSTM and CNN. Worker productivity was estimated based on the achieved accuracy results, and these estimates were compared with the actual productivity. Figure 1 illustrates the overall introduction of research.

### 2.1. Experimental Design

Recently, various developments in robots and machines that can automatically build masonry and lay bricks have emerged. For example, Brunn et al. [49] proposed scaffold-free construction of a masonry arch using a third robotic agent. Bruckmann and Boumann [50] simulated and optimized masonry construction using cable robots to stack building structure. However, masonry is an essential task for space separation and finishing. It is an indoor task performed by a small number of people (or groups of few people). Especially in apartment projects, multiple small groups of workers are concurrently distributed across a vast site. However, challenges persist in adopting robotic masonry for indoor masonry tasks similar to these. Meanwhile, compared to the work carried out in open spaces (such as rebar and formwork installations), using a vision-based approach for masonry is challenging due to budget or installation constraints. Therefore, this study conducted an experiment to measure worker productivity in masonry work using IMU sensors. Experiments were designed and conducted to collect data from an actual apartment construction project in Seongnam-si, Gyeonggi-do, South Korea. In our experiments, 30 subjects with more than 10 years of experience participated. All the subjects were medically healthy and did not have any clinical problems that affected their working performance in their masonry work. Each subject wore safety vests, gaiters, safety shoes, safety gloves, and a safety helmet. Table 1 presents the information about the subjects who participated in this study. 

**Figure 2:**
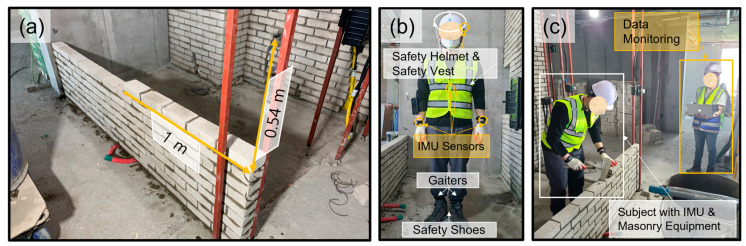
Experimental setup (image captured by the authors): (**a**) given workload, (**b**) IMU sensors attached to the subject’s body, and (**c**) data collection procedure.

The subjects were allocated with the task of stacking a brick wall (as shown in Figure 2a, 1.00 m in width and 0.54 m in height; a total of 40 bricks were given) at their own pace. The IMU sensor data were collected in real time, as shown in Figure 2b,c. 

**Figure 3:**
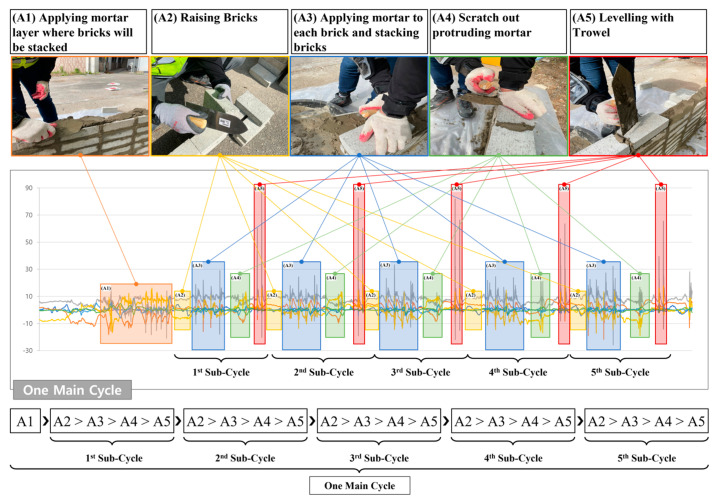
Activity and cycle classification.

Each operation in the masonry process was divided into five activities, and all other activities were considered as null values. The five activities of masonry were defined as follows (Figure 3): (A1) Application of mortar layer where the bricks will be stacked, (A2) raising of the bricks from the location they were prepared at, (A3) application of the mortar to each brick that workers pick and stack, (A4) scratching out of the protruding mortar caused by the pressure of the stacking process, and (A5) leveling of the brick with a trowel. (A1) was performed once before stacking each brick layer (each brick layer consisted of five bricks) for the efficiency of the masonry production, followed by (A2), (A3), (A4), and (A5), which were repeated in order. Since each activity needed to be unitized for productivity calculation, worker activities were divided into main and sub-cycles. The process of stacking bricks (the process of performing (A2), (A3), (A4), and (A5) in Figure 3 was defined as one sub-cycle. One main cycle was composed of performing one (A1) activity and five sub-cycles. Each subject was asked to execute eight main cycles in one experiment and repeat the experiment five times. Consequently, each subject stacked 40 bricks in each experiment and 200 bricks in total.

### 2.2. Data Processing and Data Segmentation

A total of 30 subjects participated in the experiment, and 40 bricks were stacked for each set of the experiment and a total of five sets were conducted. Therefore, the total amount of bricks piled up by all workers was 6000. The total IMU data points obtained throughout all the sets were 5,123,456. The raw data collected were processed to remove outliers in the range exceeding the maximum measurement range of the APDM sensor used in the experiment, accelerometer ±156.9 m/s^2^, and gyroscope ±2000 degree/s. Subsequently, 10 Hz 5th-order low pass Butterworth filter was applied to cut off high-frequency noise [51]. In addition, 10 Hz was set since masonry work is usually performed at less than 10 Hz [52]. Meanwhile, since IMU measures worker behavior in a continuous form, the continuous data have to be segmented into small units to distinguish each activity. This study aims to detect movements in the form of repeating periodic movements in a specific cycle. Therefore, the sliding-window segmentation was used, and each window slid every second when the data were transformed into the image [53,54,55,56]. In addition, to express all the movements without interruption, the window size for the image was set to 1490 data points, representing 11.64 s. The obtained and segmented data were labeled based on pre-determined activities by experts. 

### 2.3. Long Short-Term Memory Algorithm 

**Figure 4:**
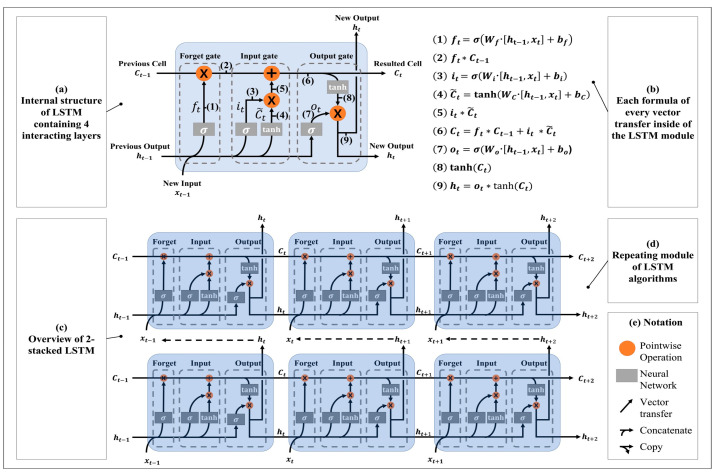
Overview of LSTM structure: (**a**) internal structure of LSTM module, (**b**) formula of every vector transfer explaining the computational mechanism of LSTM, (**c**) diagram of two-stacked LSTM layer, (**d**) repeating LSTM modules, (**e**) notations for explaining diagrams.

LSTM has been widely used for analyzing time series data as it can deal with the dividing gradient problems remarkably [44]. In our experiments, time series data of human motion were collected by an accelerometer and a gyroscope. Detailed process of LSTM is shown in Figure 4. W represented the weight metrics from new input and previous output to the hidden layer (forget gate, input gate, and output gate). The memory cell was defined as C, output cell as h, forget gate as f, output gate as o, and input gate as i. Each gate manipulated which data should be disremembered or stored as described in Figure 4a. As shown in Figure 4a, forget gate decides to overwrite by comparing the inner memory cell and new input. Input gate outputs if the data present the ongoing values to restore the LSTM cell. Finally, output gate defines if the data associate previous data or instantly appear using the data from forget gate, input gate, previous output, and new input. Input of each gate was calculated using the sigmoid function ($\sigma (x)={\left(1+e^{-x}\right)}^{-1}$) or hyperbolic tangent function. The output values of each gate within [0,1] were multiplied by pointwise operation. The computation in LSTM is detailed in Figure 4b [44,57]. Figure 4c shows the two-stacked LSTM. Two LSTM modules were arranged in parallel in two-stacked LSTM. 

### 2.4. Convolution Neural Network Algorithm 

**Figure 5:**
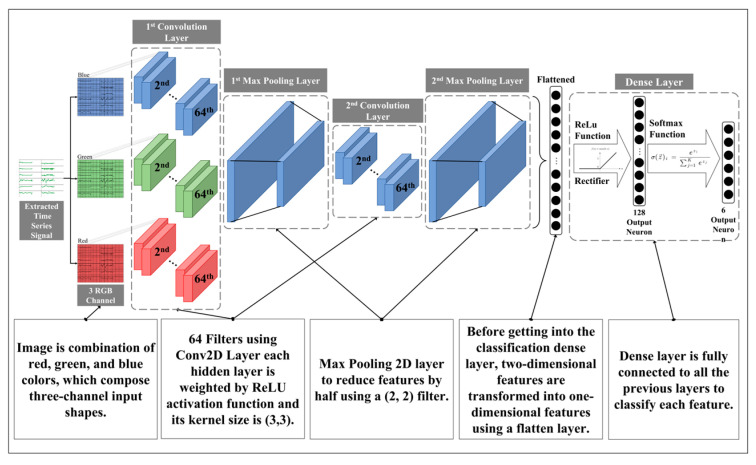
CNN structure in the case of multiple sensors.

The CNN has been studied for classifying images by extracting featured values from input data [58]. In addition, the CNN can detect a pattern that is stretched or contracted by individuality of workers. Moreover, Sadouk [59] showed that the CNN approaches can improve time series classification performance. In the process of transforming time series raw data into images, each pattern of data was set to be displayed on the same x-axis and y-axis ranges to be fitted to the same size of image. In general, CNN derives higher accuracy as images with higher resolution are obtained [60]. However, a proper level of resolution should be determined since images with higher resolution require more computational loads and resources [61]. Various levels of resolution were considered to find a proper resolution considering both computational load and accuracy. To make graph image data, the x-axis, y-axis, and z-axis of the accelerometer and the x-axis, y-axis, and z-axis of gyroscope were aligned in parallel, and each axis occupied 46 pixels vertically (the upper and lower blank space of the image were 2 pixels each). Moreover, 11.63 s of window size was represented in 300 pixels. As the number of sensors increased, the resolution increased by 300 pixels since it was listed horizontally as much as the number of sensors in the existing image. In the case of a single sensor, a pattern was based on the length of the action in a 300 × 280 pixels image. In the case of multi sensors, data collected from both wrists should be expressed; therefore, they were converted into an image with dimensions of 600 × 280 pixels to display each sensor value from the left and right wrists. Finally, in the case of using multiple sensors, data collected from both wrists, right ankle, and back of the sternum should be displayed; therefore, they were converted into a 1200 × 280 pixels image. Signals for the entire movement should be expressed in the images in order that all patterns for each action can appear in the image [62]. The overall CNN structure used in this study is illustrated in Figure 5. Two layers of Conv2D were adopted for our structure. Max pooling 2D layers, next to each Conv2D layer, reduce the number of features by half to prevent overfitting. 

We adopted the Adam optimizer in both algorithms, which is a widely used optimizer for applying different learning rates to each parameter [42,63,64,65]. We adopted the rectified linear unit (ReLU) activation function for the input and hidden layers and the softmax activation function for the output layers only in the CNN model. Owing to the characteristic of ReLU activation function [66], it has been regarded as inappropriate for LSTM. Therefore, LSTM model consists of two-stacked LSTM layers, fully connected layer, and softmax activation function. The softmax activation function is effective for observing the probability value of predicting each class. A probability value of each class is required when the results are analyzed. After model trainings, when the number of epochs was 120 or more in CNN, and 150 or more in LSTM, the accuracy of the model was stable in both models. Therefore, the epoch was empirically set to 120 for the CNN model and 150 for the LSTM model. 

### 2.5. Model Training and Performance Evaluation Methods

During the model training, the total collected data were divided into training and testing datasets. Moreover, 75% and 25% of the total dataset were used for training and testing, respectively. The training dataset was further split into two datasets, 75% of the training dataset was applied to training and the remaining 25% was applied to validation. Every step of data separation was randomly processed, and each data of every dataset was not overlapped.

For assessing performance, the performance of the suggested algorithms was evaluated by four metrics including accuracy, precision, recall, and F1-score. Accuracy stands for summarizing the classification performance of all classes. Accuracy is defined as the ratio of correct cases (sum of true positive and true negative) to all cases. Precision is calculated as the ratio of correct positive cases (true positive) to all positive cases (sum of true positive and false positive). Therefore, precision refers to exactness. Recall is the ratio of correct positive cases (true positive) to the total cases in the actual class (sum of true positive and false negative). The F1-score is the weighted average of precision and recall. Equations (1) to (4) present the calculations of each evaluation metric:

(1) $$Accuracy=\frac{TP+TN}{TP+TN+FP+FN}$$

(2) $$Precision=\frac{TP}{TP+FP}$$

(3) $$Recall=\frac{TP}{TP+FN}$$

(4) $$F1-score=2\times \frac{Precision\times Recall}{Precision+Recall}$$

### 2.6. Productivity Measurement

Organization for Economic Co-operation and Development (OECD) Manual generally defines productivity as the ratio of input volume to output volume [25,67]. Since this study aims to measure productivity based on the results of worker activity classification, the authors focus on the labor productivity [68]. Using the predicted activity through the two algorithms described in the previous section, the number of bricks stacked by the workers was calculated. The quantity of bricks was calculated in two ways. First, when (A1), which is the reference of the main cycle, was detected, it was considered that five bricks were stacked. Five bricks were counted per detection of (A1). In particular, the number of predicted (A1) times five was the number of stacked bricks. If the detecting and counting (A1) activity has a low error rate, measuring precise productivity without detailed activity classification is possible. In this case, there was no additional computational load for analyzing other activities (A2-A5). The authors call this method “A1 Calculation”. Second, if all actions that were assigned to stack one brick from (A2) to (A5) (one sub-cycle) were detected, then an algorithm counted the bricks by considering that one brick had been stacked. In this case, the number of bricks was calculated by uniting the operations (A2) to (A5) into one sub-cycle. Despite the increased computational load, it is expected to be able to derive more robust productivity. The authors call this method “Workflow Calculation”. The time was measured as the time taken when the activity predicted by each algorithm was included in (A1) to (A5). In the case of actual work, video recording was used for measuring the actual working time of the workers. In this study, productivity was calculated as the number of bricks stacked per hour. “The amount of time the worker works” and “the number of bricks the worker stacks” are information needed to measure productivity. To accurately measure these data, two cameras were used to record the sequential scenes of masonry work. One camera was installed on the side of the workers to observe the movement of the worker to verify the amount of time workers worked. Another camera was installed to observe the building of the masonry wall in progress to check the number of bricks. All cameras were measured at 60 frames per second, and the working time was calculated by checking each frame. In calculating the working time, five experts with more than 20 years of on-site construction experience inspected the recorded video and confirmed whether the work was carried out. Therefore, the individual productivity of a total of 30 workers was derived and used for comparison with automated productivity measurement results. All productivities measured through the proposed method were expressed in the number of bricks per minute. Performance of measuring productivity is presented as error rate compared to actual productivity. As a result, the relationship between the average error rate of productivity and the accuracy of activity classification is plotted to identify the tendency. 

## 3. Results

This study was conducted to confirm the validity of an automated productivity measurement approach. To implement this approach, worker activity classification was preceded by an analysis of wearable sensing data collected during masonry work. Then, the collected IMU data were used to train two deep learning models using Python for worker activity classification, and the performances of these models were calculated using a confusion matrix, precision, recall, and F1-score. The productivity measurement was performed by calculating the production volume and time utilized based on the activity classification results. The performance of productivity measurement was assessed using the error rate based on gaps between the calculated production volume and actual production volume. Finally, the error rate and the accuracy of activity classification were calculated to discuss the error rate trend as the accuracy increased. 

### 3.1. Activity Classification Performance

The accuracy, precision, recall, and F1-score according to the number of sensors and the type of the training model are presented in Table 2. As can be seen, all the accuracies obtained by the CNN exceed 90.51% and the F1-scores exceed 0.907, whereas the LSTM shows a maximum accuracy of 79.32% and a maximum F1-score of 0.820. In the case of the LSTM, the average precision was 0.724 when using a single sensor, 0.786 when using dual sensors, and 0.825 when using multiple sensors. The average recall was 0.742 when using a single sensor, 0.794 when using dual sensors, and 0.814 when using multiple sensors. In the case of the CNN, average precision was 0.903 when using a single sensor, 0.928 when using dual sensors, and 0.963 when using multiple sensors. The average recall is 0.912 when using a single sensor, 0.941 when using dual sensors, and 0.970 when using multiple sensors. All the average precision and recall values increased with the increase in the number of sensors and when the CNN was used. The best classification results obtained using the LSTM (79.32% accuracy and 0.820 F1-score with multiple sensors) and worst classification results obtained using the CNN (90.51% accuracy and 0.907 F1-score when using a single sensor) have a gap of more than 10% in terms of both the F1-score as well as the accuracy; this is the largest performance difference between the models.

All the null values showed the lowest precision and recall values for each activity, except for the (A2) recall value (0.642, which is lower than null (0.700)) when using dual sensors with the LSTM and (A2) recall value (0.670, which is lower than null (0.771)) when using multiple sensors with the LSTM. In contrast to the activities of (A1) to (A5), which were performed based on similar movements, the null showed the lowest precision and recall in most of the activity classification results since there was no representative movement. 

Consequently, the activity classification accuracy and F1-score were higher for the CNN than for the LSTM, and the performance metrics were higher when the number of sensors increased. Additionally, the accuracy and F1-score of using multiple sensors with the LSTM (accuracy of 79.32% and F1-score of 0.820) did not exceed the accuracy when using the CNN with a single sensor (accuracy of 90.51% and F1-score of 0.907). When the number of sensors increased, the average increase in the accuracy of the LSTM model was 4.91%, whereas the CNN showed an average increase of 3.37%. However, when the training model was changed from LSTM to CNN, the increase in accuracy for each sensor was 17.61%p (percentage point) on average. 

This study divided the masonry process into five detailed activities. From the activity classification results, the average duration of the (A2), (A4), and (A5) activities were shorter than the average duration of (A1) and (A3) activities. Overall, the CNN showed higher precision and recall values than the LSTM. However, the precision values for all combinations in the LSTM model except for the case with a single sensor were similar or greater than those of the CNN model in the (A1) and (A3) activities, which were longer than the other activities. Table 2 summarizes the classification evaluation metrics.

**Figure 6:**
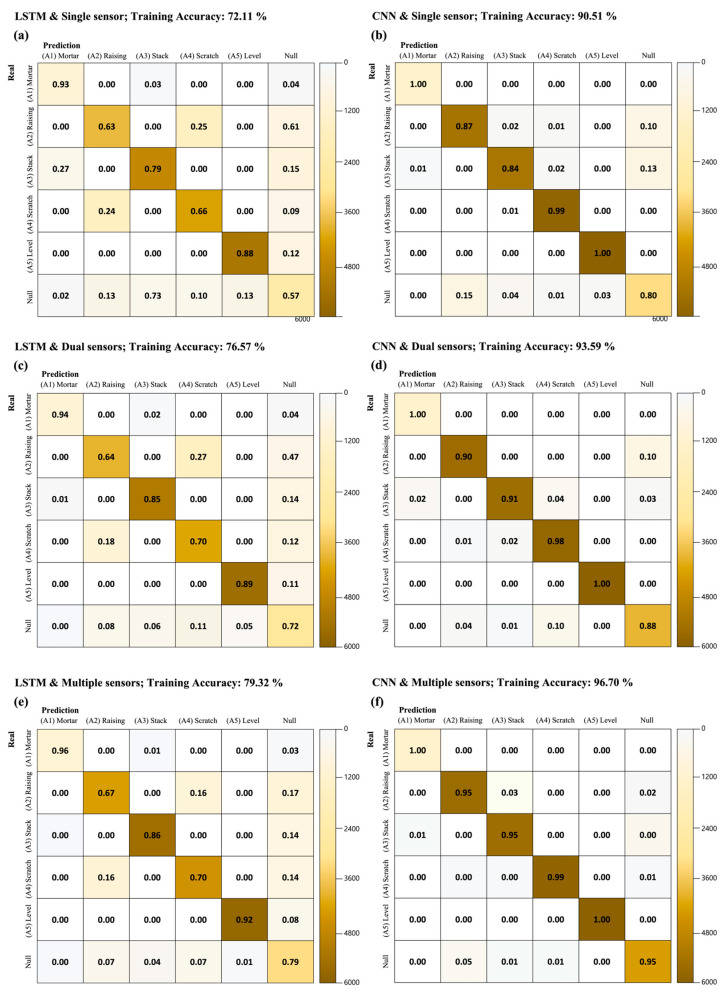
Results of activity classification: (**a**) multi-class confusion matrix using single sensor and LSTM using testing dataset, (**b**) multi-class confusion matrix using single sensor and CNN using testing dataset, (**c**) multi-class confusion matrix using dual sensors and LSTM using testing dataset, (**d**) multi-class confusion matrix using dual sensors and CNN using testing dataset, (**e**) multi-class confusion matrix using multiple sensors and LSTM using testing dataset, (**f**) multi-class confusion matrix using multiple sensors and CNN using testing dataset.

Figure 6 illustrates the confusion matrices for the different models and the collected dataset. Figure 6a,c,e present the confusion matrices when using single sensors, dual sensors, and multiple sensors, respectively, derived using the LSTM. Figure 6b,d,e present the confusion matrices when using the single sensor, dual sensors, and multiple sensors, respectively, derived through the CNN model. As presented in Table 2, the training accuracy increased with the increase in the number of sensors, and an increase in the training accuracy when the model change was carried out was higher than that when the number of sensors were increased. Thus, diagonal concentration resulting from horizontal comparison (i.e., Figure 6a–f) is more pronounced than resulting from vertical comparison (i.e., Figure 6a–f). 

### 3.2. Productivity Measurement

**Figure 7:**
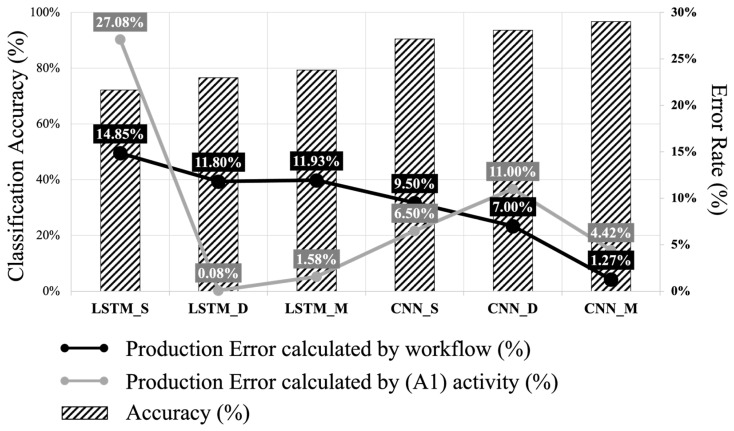
Activity classification accuracy and error rate of production prediction.

In this study, the productivity is expressed as the number of bricks stacked per minute. The method of measuring production was divided into two parts (i.e., (A1) Calculation and Workflow Calculation) and the error for each part was calculated. Figure 7 shows the activity classification accuracy (bar graph), the production error for the Workflow Calculation (black line), and the production error for the (A1) Calculation (gray line). The highest production error (27.08%) was derived from the (A1) Calculation and the LSTM single sensor had the lowest accuracy (72.08%). While the production error of the LSTM dual sensors shows the second lowest accuracy (76.57%), production error was the lowest with 0.08%. Although the production was measured using activity classification accuracy, (A1) Calculation does not show the correlation trend with activity classification accuracy. Therefore, productivity measurement using (A1) Calculation is less effective to derive significant results in automated productivity measurement. Meanwhile, production error with Workflow Calculation begins with 14.85% at LSTM_S and ends with 1.27% at CNN_M. The trend of the Workflow Calculation shows that the error decreases as the activity classification accuracy increases, except when there is a transition from LSTM_D (11.80%) to LSTM_M (11.93%). The analysis was executed using Workflow Calculation for measuring production in automated productivity measurement since it shows correlation tendency to the error and activity classification accuracy.

**Figure 8:**
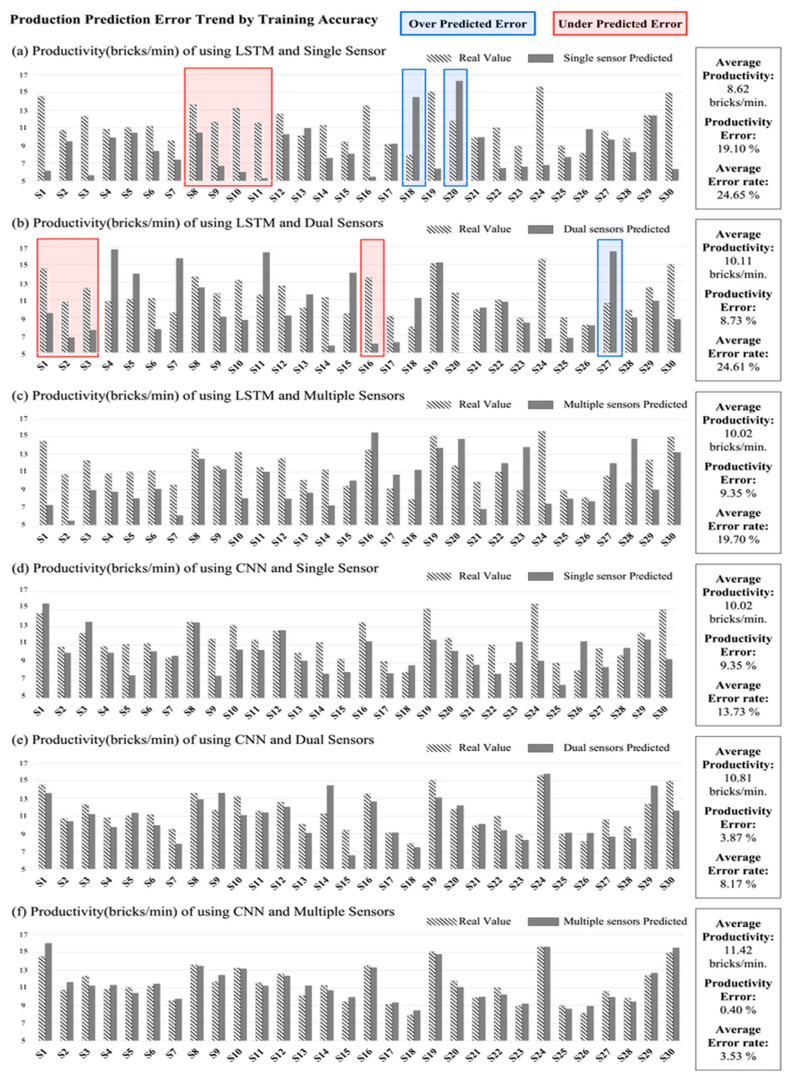
Predicted production and individual productivity with different numbers of sensors and algorithms: (**a**) comparison of productivity (bricks/min) with LSTM and single sensor, (**b**) comparison of productivity (bricks/min) with LSTM and dual sensors, (**c**) comparison of productivity (bricks/min) with LSTM and multiple sensors, (**d**) comparison of productivity (bricks/min) with CNN and single sensor, (**e**) comparison of productivity (bricks/min) with CNN and dual sensors, (**f**) comparison of productivity (bricks/min) with CNN and multiple sensors.

The average actual productivity from measuring the actual working time in recorded videos was 11.36 bricks/min. Figure 8 shows the comparison between the productivity measurement results based on Workflow Calculation and actual productivity by workers. Striped bar indicates the actual productivity by workers. Average productivity represents the average predicted productivity of each worker, and the productivity error represents the error between actual and predicted average productivity. Productivity error was calculated for acquiring information about productivity, which is the aim of this study. Average error rate indicates the percentage of the absolute value of the difference between actual and predicted productivity divided by the actual productivity. The highest productivity error was 19.10% in LSTM for single sensor usage. The lowest productivity error was derived from CNN for multiple sensors, which was 0.04%. 

When switching the number of sensors from dual sensors to multiple sensors (Figure 8b,c), the error slightly rises to about 0.62%p. When changing the number of sensors and algorithms from LSTM multiple sensors to CNN single sensor (Figure 8c,d), the errors were maintained at 9.35%. However, the error rate drops to 3.87%p in Figure 8e. In calculating productivity error, the decreasing tendency in accordance with the increase in activity classification accuracy is not remarkably observed. This tendency can be explained by underpredicted productivity (red boxes in Figure 8a,b) and overpredicted productivity (blue boxes in Figure 8a,b). Productivity errors represent the difference between average predicted productivity and actual productivity. When the predicted productivity is higher than the averages, productivity error has a positive value which is the blue box group in Figure 8a,b. Moreover, when the predicted productivity is lower than the average, productivity error has a negative value, which is the red box group in Figure 8a,b. Consequently, the red and blue box groups countervail each other. Meanwhile, the average error rate using the absolute value of each error reflects the degree of countervailed error from the productivity error.

The average error rate was the highest at 24.65% (Figure 8a) and the lowest at 3.53% (Figure 8f). The average difference in the average error gap of each step of Figure 8a–f is 4.224%p. A comparison between Figure 8c,d shows the largest difference (5.97%p) in the average error rate. The difference in activity classification accuracy in Figure 8c,d is also the largest (11.19%p), far exceeding the average difference in accuracy (4.92%p). These results indicate that the classification accuracy and productivity prediction error are inversely related. 

## 4. Discussions

### 4.1. Underpredicted and Overpredicted Errors in Productivity Calculation

This section discusses productivity measurement when the accuracy of activity classification is low. As a result, the gap with the actual productivity and calculated productivity may increase when the model has low accuracy. Low accuracy primarily results from two types of errors: overpredicted errors and underpredicted errors. These two types of errors may cancel each other out during the measurement of average productivity and affect the evaluation of average productivity. Furthermore, these two types of errors have an inverse relationship with the accuracy of activity classification. In the case of underpredicted errors, movements of subjects that contribute to productivity are considered as null values. The individuality of the subjects also affects the productivity prediction results. The peak value of the signal pattern is lower than the average peak value of the other subjects (for example, the strength of movements is softer than the other subjects). Some subjects move faster than others in specific tasks (for example, subject 11 performed (A2) and (A3) faster than the other subjects). 

However, when the subjects performed their tasks at a slower pace, the deep learning model often considered (A5) as (A1). Additionally, the null value was predicted as (A1) to (A5) when the movement patterns for the null value and those for a productivity contributing activity were similar. These incorrect predictions can lead to the overpredicted error. However, since productivity was measured using workflow calculation, it was difficult to count as the production even when the movements for a productivity contributing activity were considered as null values. Therefore, these errors caused an increase in the production time rather than an increase in production amount, and represented underpredicted errors; therefore, these errors are referred to as underpredicted errors. Consequently, the underpredicted errors occurred in a larger proportion than the overpredicted errors. However, these errors can be prevented by increasing the number of sensors and optimizing the algorithms by providing complementary information and finding suitable training models. 

### 4.2. Accuracy Comparison between Different Algorithms and Research

Activity recognition plays an important role in many research fields as it can detect movements and provide a high level of knowledge about human activity [69]. Research on activity recognition has been conducted in various research fields including the construction industry. In this discussion, the authors compare the results of this study with the accuracy reported in: (1) other studies that used deep learning algorithms (such as the LSTM and CNN), (2) other studies on activity recognition in masonry work. Additionally, the performance of the various RNN and CNN algorithms is compared to the main algorithms of this study. The comparison results provide a robust justification of the selection of algorithms in this study.

Ronao and Cho [70] investigated activity recognition performance of six different daily life activities (such as walking, walking upstairs, walking downstairs, sitting, standing, and laying) using wearable sensors. They achieved 95.75% accuracy using a CNN and temporal fast Fourier transform of the HAR dataset. In their study, the wearable sensors were placed in the pockets of the subjects, which resulted in unexpected noise. Hence, data may have contained unnecessary movements that were recorded during the measurement process. While the above study put the sensors in the pocket, the sensor was firmly fixed to the subjects during the experiment in authors’ study. The proper attachment of sensors to the subjects may derive higher accuracy in their study. 

Mahmud et al. [71] used the PhysioNet database [72], which contains data from PPG, ECG, and motion sensors. In their study, PPG and motion sensor data were selectively used for activity recognition (such as walking, running, low and high resistance biking). Their study used a multi-stage LSTM and achieved an average F1-score of 83.9% and accuracy of 83.2%. The results of these studies can be further improved through the use of multiple sensors that can provide complementary information for improving accuracy [43,73]. Consequently, compared to the present study, the accuracy of the study reported using the CNN-based approach is slightly lower and the study reported using the LSTM-based approach is higher.

Meanwhile, Ryu et al. [22] divided masonry work into four different activities (spreading mortar, laying blocks, adjusting blocks, and removing mortar) and classified each activity by using different approaches. They selected four different features (namely, k-nearest neighbors, multilayer perceptron, decision tree, and multiclass SVM) and four different window sizes (1–4 s) for analyzing the activities. In their study, the accuracy ranged from 82.9% to 88.1% depending on the type of classifier used with the 4 s of window. Their study used machine learning approaches for extracting the activities, in contrast to the current study that uses deep learning approaches. 

Moreover, in the current study, the authors attempted to justify the objectivity by comparing other algorithms. Therefore, two recurrent neural network (RNN) [74] (RNN and deep recurrent neural network (DRNN) [75,76])-based and two CNN (LeNET [77,78] and AlexNET [79,80])-based approaches were applied to the dataset used in this study. Overall, it was found that the results derived using the main algorithms (LSTM and CNN) were similar to those derived using the compassion algorithms. The results also showed that CNN-based approaches have higher accuracy and F1-score as compared to RNN-based approaches. Particularly, in RNN-based approaches, the maximum F1-score was 0.703 when using the DRNN with multiple sensors. The RNN did not seem to be suitable for the dataset. On the other hand, AlexNET with multiple sensors achieved an accuracy of 94.75% and F1-score of 0.857. Therefore, the CNN-based approaches appeared to achieve a desirable level of accuracy in detailed activity classification. Although the activity recognition results using AlexNET showed a desired level of performance, as mentioned previously, a model with high accuracy and F1-score is needed for detailed activity detection. Table 3 represents the performance evaluation results of activity classification using various algorithms.

### 4.3. Contributions and Practical Applications

The main contributions of this study are as follows: (1) worker productivity information on an individual level could be acquired through detailed activity classification, (2) an optimal algorithm for IMU-based deep learning activity recognition could be selected by comparing and analyzing the various deep learning algorithms, (3) information on how many sensors are needed for an appropriate level of activity recognition was provided by verifying the difference in accuracy according to the number of sensors, and (4) on-site human resource management can be improved by measuring individual productivity. 

As shown in the productivity measurement section, the necessity of detailed activity measurement was confirmed through the results that showed that productivity measurement based on detailed activity derives high accuracy. Although the model training time took about 137 min, the process of identifying worker activity was able to determine in a very short time of about 2 s. These results can be used as basic data for developing novel human resource management for masonry work. Individual productivity measured automatically can contribute to the efficient allocation of manpower. Additionally, if individual productivity data supplemented by additional information (e.g., location data) are applied to building information modeling (BIM), real-time progress can be checked. Moreover, we noted the stable pricing trend of inertial measurement unit (IMU) sensors. Technological advancements, falling raw material costs, and consistent supply have contributed to a stable cost for IMU sensors. This stability suggests that the initial investment in sensor acquisition will likely yield a favorable cost-to-benefit ratio over the long term. 

This study also contributes to confirming activity classification accuracy according to the number of sensors attached to workers. In industrial aspects, this study can be used as an indicator of field application to determine the appropriate number of sensors. Moreover, detailed activity classification is expanded to other tasks (e.g., rebar, tile work, and form work, etc.). 

### 4.4. Limitations and Future Research

The limitation of this study can be summarized as follows:

(1) Field Applicability.
(2) Accuracy of general level of sensors.
(3) Proper fixation of sensors.

The proposed approach is focused on masonry work, which may lead to applicability issues while applying to other fields. To address this problem, worker activity data from other tasks such as rebar and carpentry work must be collected and analyzed. By increasing the applicability in the actual field by analyzing various types of work (e.g., rebar, carpentry work, form work, etc.), the versatility and utility of IMU-based activity recognition can be confirmed from the industrial aspect. Additionally, the sensors used in this study for collecting worker body movements are highly accurate with laboratory-level accuracy. The size of the sensors also did not act as a hindrance to the activity of the worker; however, it may not always be possible to use these sensors due to budget constraint. Therefore, methods of using wearable devices such as smart watches could be implemented. In this case, low-cost sensors may require additional verification of whether data are measurable to a reliable level. Meanwhile, in the case of actual field application, improper fixation may occur in the process of wearing the sensor. In these cases, the unexpected noise can occur as the sensor may not fully reflect the movement of the workers. Therefore, additional verification of the activity recognition performance may be required with data when the sensor is improperly attached to the body.

Consequently, the authors recognize the significance of assessing the long-term performance of the proposed approach and securing the availability of the proposed approach. More specifically, regarding the limitations we presented, the authors plan to explore alternative sensor configuration or various task availabilities.

## 5. Conclusions

This study investigated the feasibility of automated productivity measurement via sensor-based activity classification. This study hypothesized that the accuracy of activity classification differs depending on the number of sensors and the type of algorithms used. The data collected from the experiments by varying the number of sensors were used to train an LSTM and CNN. The method of estimating production by extracting workers’ activities (i.e., (A1) Calculation) exhibited low reliability in terms of the error rate. Therefore, a Workflow Calculation method was applied for measuring production. This method can decrease the error rate of productivity measurement. Furthermore, the productivity error was below 10% when using CNN and dual sensors, demonstrating the potential for accurately measuring productivity using a small number of IMU sensors. 

Consequently, it was found that the two deep learning algorithms (i.e., CNN and LSTM) used in this study can effectively classify workers’ activities in masonry. Furthermore, it was noted from the results of the activity classification that the number of sensors affects the accuracy of the classification. Based on the activity classification results, the feasibility of an automated framework for measuring productivity through activity classification was demonstrated. 

This study identified the necessity of classifying detailed worker activity for calculating production. It was also found that extracting some workers’ activities increased the possibility of errors when measuring productivity. This finding can be a basis for improving the developed automated productivity measurement method using IMU sensors. However, its results are restrictedly shown in masonry task. Other tasks should be validated by future research to ensure the robustness of the current research.

## Figures and Tables

**Table 1:** Subject information.

	Height (cm)	Weight (kg)	Age (Years)
Mean	174.66	71.18	40.40
Median	173.77	71.56	40
Standard Deviation	5.57	8.68	7.02
Min Value	163.48	58.52	29
Max Value	182.52	90.89	52

**Table 2:** Evaluation of activity classification using LSTM and CNN.

**LSTM Results**																		
	**Single Sensor**						**Dual Sensors**						**Multiple Sensors**					
Training Accuracy	0.7208 (72.08%)						0.7657 (76.57%)						0.7932 (79.32%)					
Precision	A1	A2	A3	A4	A5	Null	A1	A2	A3	A4	A5	Null	A1	A2	A3	A4	A5	Null
	0.734	0.649	0.931	0.674	0.905	0.449	0.938	0.728	0.945	0.669	0.964	0.518	0.975	0.761	0.958	0.747	0.994	0.518
Avg. prec.	0.724						0.786						0.825					
Recall	A1	A2	A3	A4	A5	Null	A1	A2	A3	A4	A5	Null	A1	A2	A3	A4	A5	Null
	0.933	0.626	0.793	0.662	0.879	0.558	0.939	0.642	0.847	0.698	0.889	0.700	0.958	0.670	0.861	0.701	0.925	0.771
Avg. recall	0.742						0.794						0.814					
F1-score	0.732						0.790						0.820					
**CNN results**																		
	**Single Sensor**						**Dual Sensors**						**Multiple Sensors**					
Training Accuracy	0.9051 (90.51%)						0.9359 (93.59%)						0.9670 (96.70%)					
Precision	A1	A2	A3	A4	A5	Null	A1	A2	A3	A4	A5	Null	A1	A2	A3	A4	A5	Null
	0.939	0.892	0.929	0.967	0.982	0.708	0.901	0.965	0.976	0.902	0.999	0.825	0.958	0.960	0.962	0.990	1.000	0.910
Avg. prec.	0.903						0.928						0.963					
Recall	A1	A2	A3	A4	A5	Null	A1	A2	A3	A4	A5	Null	A1	A2	A3	A4	A5	Null
	1.000	0.865	0.841	0.986	1.000	0.780	1.000	0.897	0.913	0.976	1.000	0.860	1.000	0.954	0.950	0.985	1.000	0.929
Avg. recall	0.912						0.941						0.970					
F1-score	0.907						0.935						0.967					

**Table 3:** Evaluation of activity classification using various algorithms.

**Recurrent** **Algorithms**	**RNN** **Single**	**RNN** **Dual**	**RNN** **Multiple**	**DRNN** **Single**	**DRNN** **Dual**	**DRNN** **Multiple**	**LSTM** **Single**	**LSTM** **Dual**	**LSTM** **Multiple**
Accuracy (%)	71.55	74.07	76.13	71.67	74.59	77.05	72.08	76.57	79.32
AVG. Recall	0.569	0.598	0.629	0.659	0.671	0.709	0.742	0.794	0.814
AVG. Precision	0.571	0.586	0.636	0.641	0.667	0.698	0.724	0.786	0.825
F1-Score	0.570	0.592	0.632	0.650	0.669	0.703	0.732	0.790	0.820
**Convolution** **Algorithms**	**LeNET** **Single**	**LeNET** ** Dual**	**LeNET** **Multiple**	**AlexNET** **Single**	**AlexNET** **Dual**	**AlexNET** **Multiple**	**CNN** **Single**	**CNN** **Dual**	**CNN** **Multiple**
Accuracy (%)	86.24	90.05	93.35	88.04	91.64	94.75	90.51	93.59	96.70
AVG. Recall	0.724	0.734	0.796	0.754	0.810	0.851	0.912	0.941	0.970
AVG. Precision	0.719	0.741	0.848	0.776	0.797	0.864	0.903	0.928	0.963
F1-Score	0.721	0.737	0.821	0.765	0.803	0.857	0.907	0.935	0.967

## Data Availability

All data, models, or code generated or used during the study are available from the corresponding author by request.
